# The Antiplatelet Effect of 4-Methylcatechol in a Real Population Sample and Determination of the Mechanism of Action

**DOI:** 10.3390/nu14224798

**Published:** 2022-11-13

**Authors:** Marcel Hrubša, Lukáš Konečný, Markéta Paclíková, Mst Shamima Parvin, Pavel Skořepa, František Musil, Jana Karlíčková, Lenka Javorská, Kateřina Matoušová, Lenka Kujovská Krčmová, Alejandro Carazo, Alena Šmahelová, Vladimír Blaha, Přemysl Mladěnka

**Affiliations:** 1 The Department of Pharmacology and Toxicology, Faculty of Pharmacy in Hradec Králové, Charles University, 50005 Hradec Kralove, Czech Republic; 2 The 3rd Department of Internal Medicine-Metabolic Care and Gerontology, University Hospital and Faculty of Medicine in Hradec Králové, Charles University, 50005 Hradec Kralove, Czech Republic; 3 The Department of Pharmacognosy and Pharmaceutical Botany, Faculty of Pharmacy in Hradec Králové, Charles University, 50005 Hradec Kralove, Czech Republic; 4 The Department of Military Internal Medicine and Military Hygiene, Faculty of Military Health Sciences, University of Defence, 50001 Hradec Kralove, Czech Republic; 5 The Department of Occupational Medicine, University Hospital and Faculty of Medicine in Hradec Králové, Charles University, 50005 Hradec Kralove, Czech Republic; 6 The Department of Clinical Biochemistry and Diagnostics, University Hospital Hradec Králové, 50005 Hradec Kralove, Czech Republic; 7 The Department of Analytical Chemistry, Faculty of Pharmacy in Hradec Králové, Charles University, 50005 Hradec Kralove, Czech Republic

**Keywords:** platelet, flavonoid, metabolite, aggregation, human, blood

## Abstract

A polyphenol-rich diet has beneficial effects on cardiovascular health. However, dietary polyphenols generally have low bioavailability and reach low plasma concentrations. Small phenolic metabolites of these compounds formed by human microbiota are much more easily absorbable and could be responsible for this effect. One of these metabolites, 4-methylcatechol (4-MC), was suggested to be a potent anti-platelet compound. The effect of 4-MC was tested ex vivo in a group of 53 generally healthy donors using impedance blood aggregometry. The mechanism of action of this compound was also investigated by employing various aggregation inducers/inhibitors and a combination of aggregometry and enzyme linked immunosorbent assay (ELISA) methods. 4-MC was confirmed to be more potent than acetylsalicylic acid on both arachidonic acid and collagen-triggered platelet aggregation. Its clinically relevant effect was found even at a concentration of 10 μM. Mechanistic studies showed that 4-MC is able to block platelet aggregation caused by the stimulation of different pathways (receptors for the von Willebrand factor and platelet-activating factor, glycoprotein IIb/IIIa, protein kinase C, intracellular calcium elevation). The major mechanism was defined as interference with cyclooxygenase-thromboxane synthase coupling. This study confirmed the strong antiplatelet potential of 4-MC in a group of healthy donors and defined its mechanism of action.

## 1. Introduction

The current antiplatelet therapy is facing many issues that complicate the prevention and treatment of thromboembolic conditions. Acetylsalicylic acid (ASA), the most commonly prescribed drug in secondary prevention, is often met with insufficient response, now also known as “aspirin resistance”, which limits its use [1]. Other drugs developed with the intention of interfering with the arachidonic acid cascade of platelet aggregation have not yet produced any significant additional profit. Different available options, such as antagonists of ADP receptor P2Y_12_, are efficient but face other issues. An older-generation drug, clopidogrel, is a pro-drug with a great deal of variability in response due to inter-individual differences in biotransformation. Newer drugs, such as prasugrel and ticagrelor, are not burdened by these shortcomings, but the former is associated with a higher risk of bleeding, while the latter is prone to other pharmacokinetic interactions [2]. There is also a relatively novel drug, vorapaxar, an antagonist at the thrombin PAR1 receptor, which is, however, currently not available in Europe [3]. The remaining options, glycoprotein IIb/IIIa antagonists, are limited by their parenteral administration and high potency with an elevated risk of bleeding, which restricts their use to only acute interventions for in-hospital settings. Moreover, the resistance to antiplatelet drugs can be more relevant in some pathological conditions. In particular, patients suffering from diabetes mellitus represent a special condition where a high degree of resistance to current antiplatelet drugs has been observed [4,5,6]. Therefore, the search for novel antiplatelet drugs that circumvent the aforementioned issues is still highly relevant.

Our group has recently shown that one metabolite of flavonoids, a common part of our diet, has strong antiplatelet effects and is about one order of magnitude more potent than ASA [7]. This metabolite, 4-methylcatechol (4-MC), has also attracted attention due to other potential biological properties. Animal studies have confirmed the effect of 4-MC in a plethora of models. 4-MC has clearly ameliorated neurotoxicity and kidney damage and was shown to be protective of β-cells. With regards to neural tissue, a protective effect of 4-MC has been observed in models representing neuropathy, depression, pain perception and recovery after injury to both motor and perceptive neuronal tissues [8,9,10,11]. However, the mechanism of action by which these effects are exerted is not clear. In our previous work, we showed that inhibition of neither cyclooxygenase 1, nor thromboxane synthase, is the primary mechanism of action. In relation to its antiplatelet potential, it has only been indicatively determined to be based on interaction with the calcium signaling pathway [7].

4-MC represents a point of intersection between drug research and the potentially positive effects of diet on human health. In fact, this compound could be responsible, potentially with other small phenolic metabolites, for the claimed positive effects of diets rich in polyphenols. Indeed, various studies demonstrated the beneficial effects of such diets on cardiovascular health [12,13]. It should also be mentioned that 4-MC reaches much higher concentrations in plasma than the parent flavonoids. Although such studies are limited, one paper reported a maximal concentration of 3.5 μM of 4-MC sulfate after administration of cranberry juice [14]. Hence, fairly high plasma concentrations are achievable merely by dietary intake, suggesting that the bioavailability of this compound is sufficient to achieve a pharmacologic effect. Even though studies with ileostomized patients have shown that human gut microbiota is necessary to generate flavonoid metabolites in sufficient quantities, the oral bioavailability of 4-MC in an appropriate drug formulation is likely [14,15,16,17]. Last but not least, 4-MC, given its natural origin, would also represent an acceptable drug for patients who refuse synthetic drugs.

This publication had two main aims: (1) to confirm the antiplatelet effect of 4-MC utilizing an age-heterogenous and larger population sample of generally healthy adult volunteers and (2) to investigate the mechanism of its antiplatelet action.

## 2. Materials and Methods

### 2.1. Chemicals

Dimethyl sulfoxide (DMSO), ristocetin (ristomycin monosulfate), ethylenediaminetetraacetic acid disodium salt (EDTA), platelet-activating factor-16 (PAF), acetylsalicylic acid (ASA), 4-methyl-1,2-benzenediol (4-methylcatechol, 4-MC), 9,11-dideoxy-9α,11α-methanoepoxy prostaglandin F2α (U-46619) and ticagrelor were purchased from Sigma (St. Louis, MO, USA). Arachidonic acid (AA), adenosine-5’-diphosphate (ADP) and thrombin receptor agonist peptide-6 (TRAP) were obtained from Roche (Basel, Switzerland). The physiological solution was purchased from B. Braun (Melsungen, Germany), and collagen from Diagnostica, a.s. (Prague, Czech Republic). Vorapaxar was purchased from Selleck Chemicals GmbH (Munich, Germany).

### 2.2. Methodology

#### 2.2.1. Donor Criteria

Adult volunteers (aged 20–66 years old) were selected to simulate a wider population sample of the generally healthy population. The inclusion criteria were: age ≥ 20 years and subjectively reporting good health. Exclusion criteria were the presence of heart disease, diabetes mellitus of both types, peripheral artery disease, a history of stroke, and the use of drugs that directly affect platelet aggregation (antiplatelet drugs, NSAID). Patients with diagnosed hyperlipidemia were also not recruited, but as part of a concurrent biochemical analysis, 2 patients with newly diagnosed mild hypercholesterolemia were included. Smoking, the administration of oral contraception and controlled hypertension were acceptable for enrolment in this study in order to enrich the variability of the group. Alcohol was not allowed 24 h prior to blood collection since ethanol is known to have an effect on platelet aggregation. All donors signed informed consent in line with the approvals of the ethics committees of the University Hospital in Hradec Králové (No. 201907 S04P from 21 June 2020 and No. 202007 S01P from 18 June 2020) and of the Faculty of Pharmacy in Hradec Králové (approval date: 31 May 2019). All experiments conformed to the latest Declaration of Helsinki. Data on weight and height were used for calculating body mass index (BMI) using the known formula. A mandatory questionnaire was filled in by every patient, along with their signed informed consent.

#### 2.2.2. Blood Collection

Blood samples from a total of 53 human donors were collected by venipuncture into plastic disposable syringes containing either heparin sodium (170 IU/10 mL, for platelet aggregation experiments) or with a clotting accelerator (for making a biochemical assessment of basic metabolic parameters in serum). Blood collection was always performed in the morning (7:30–8:30 a.m.). The blood was carefully transferred to the laboratory for analysis. For mechanistic experiments, an additional 14 healthy donors were recruited.

#### 2.2.3. Blood Processing

Heparinized blood was gently and slowly mixed after arrival at the faculty and placed in a designated container.

For some of the mechanistic experiments, platelet-rich plasma (PRP) was prepared by centrifuging whole blood at 214× *g* for 8 min (VWE compact Star CS4 centrifuge, VWR International Ltd., Lutterworth, UK). Afterward, the supernatant was collected, and the remaining sample was once again centrifuged, this time at 2800× *g* for 10 min to obtain platelet-poor plasma. Platelet number was counted via a Neubauer Improved counting chamber (Marienfeld, Lauda-Königshofen, Germany) with the use of an XDS-1R inverted microscope (Optica, Ponteranica, Italy). Platelet concentration was adjusted to 3.5 × 10^8^ per milliliter using platelet-poor plasma. 

#### 2.2.4. Biochemical Assessment of Creatinine 

Creatinine was measured in both the serum and urine. Analysis was carried out using a Prominence LC 20 HPLC set with an SPD-M20A Shimadzu (Kyoto, Japan) diode array detector. As the stationary phase, two monolithic columns RP-18e (4.6 mm × 50 mm, 3.0 mm × 100 mm) were connected together in combination with a 15 mmol/L phosphate buffer as the mobile phase. Creatinine was detected at 235 nm using diode array detection [18].

#### 2.2.5. Aggregometry—Group Study

The experiments were initiated precisely 30 min after the blood draw. Briefly, 300 µL of whole blood were diluted with an equal volume of preheated 0.9% sodium chloride and incubated with 5 µL of 4-MC, the standard drug or DMSO (the solvent) at a final concentration of 0.8% for 3 min at 37 °C. Platelet aggregation was then induced with one of the following inducers at the desired final concentrations (Table 1) and monitored for 6 min by use of an impedance aggregometer Multiplate (Roche Diagnostic, Basel, Switzerland). Described aggregation experiments were always performed according to a pre-established protocol with constant time intervals. 

#### 2.2.6. Mechanistic Experiments 

Whole human blood or PRP was treated with either 4-MC or the solvent and induced to aggregate by adding an inducer at the following concentration ranges: PAF (20–80 nM), a protein kinase C activator bryostatin-1 (8–80 nM), an inhibitor of sarco/endoplasmic reticulum Ca^2+^-ATPase (SERCA) thapsigargin (20–800 nM), collagen (0.16–2.42 μg/mL), arachidonic acid (25–200 μM), ristocetin (300–400 μM) and an activator of glycoprotein receptor IIb/IIIa dithiothreitol (410 μM). The reaction was followed as in the previous case for 6 min with the aggregometer Multiplate.

##### Thromboxane Synthase Assay

This assay was performed according to a previously described protocol [7]. The collected blood was immediately mixed with a terutroban solution (at a final concentration of 2 µM) to block the aggregation of platelets mediated by formed thromboxane A_2_, and PRP was prepared as described in Section 2.2.3.

This platelet suspension was incubated with the tested compounds (4-MC, ASA or a known inhibitor of thromboxane synthase 1-benzylimidazole) for 3 min at 37 °C. After incubation, the reaction was started by adding AA at a final concentration of 100 µM. The reaction was terminated precisely after 7 min by adding 500 µL of cold (4 °C) 2 mM solution of EDTA. After centrifugation at 10,500× *g* (MPW-52 centrifuge, MPW Med. Instruments, Warsaw, Poland), the levels of thromboxane B_2_, a stable metabolite of unstable thromboxane A_2,_ were measured using a commercial kit from Cayman Chemical Company (Thromboxane B_2_ Express ELISA kit-Monoclonal (Ann Arbor, MI, USA)) [19].

#### 2.2.7. Statistical Analysis

GraphPad Prism 9.3.1 (GraphPad Software, San Diego, CA, USA) was used for making all data analyses. The differences were analyzed by ANOVA followed by Dunnett’s multiple comparisons test or the Student’s *t*-test, depending on the number of variables. Correlations were tested by Pearson’s correlation test. In the case of significant correlations, linear regression was also performed.

## 3. Results

In order to confirm our previous results related to the antiplatelet effects of 4-MC observed in a limited number of healthy young adults [7], a group of overall healthy donors of a wide age range without any cardiac diseases or diabetes mellitus was selected. Our group was composed of 27 men and 26 women with a median age of 44 years. We did not exclude donors with other illnesses that were considered minor and with no direct effect on platelet aggregation. The list of these diseases and their frequency is shown in Supplementary Materials Table S1, with the corresponding medication in Supplementary Materials Table S2. The most common diagnosis was hypertension (10 donors, 19%), and the most frequent drug class was antihistamines (eight donors, 15%). Initially, we aimed to have patients solely without any hyperlipidemia, but during the study, it was revealed that two of these donors (4%) without a previous history of hyperlipidemia were found to be mildly hypercholesterolemic on the day of testing. Furthermore, 13 donors were active smokers (25%), and 18 (34%) had previously been diagnosed with COVID-19. 

**Figure 1:**
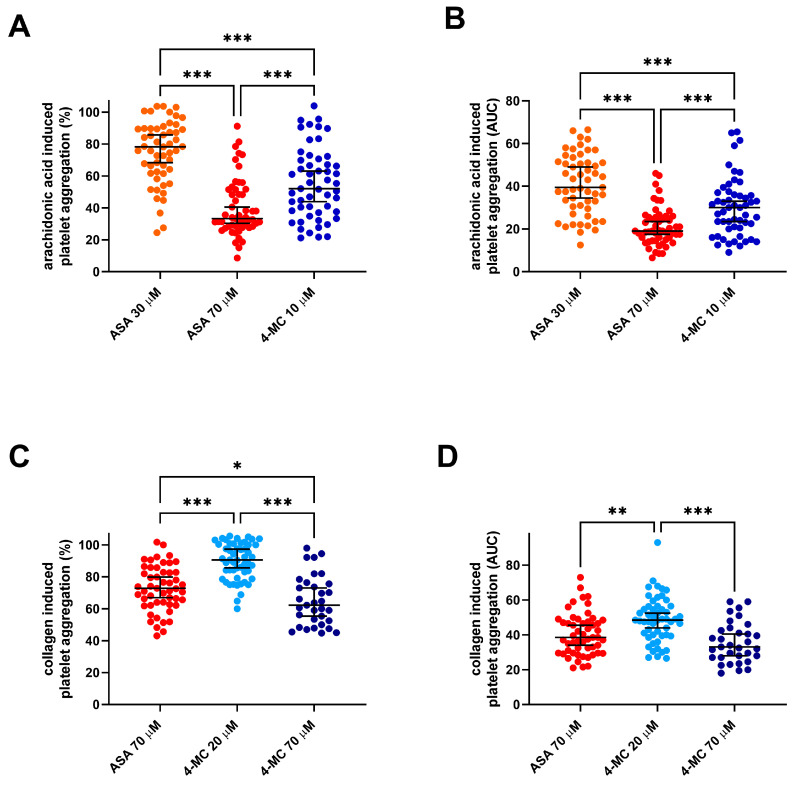
A comparison of the antiplatelet effects of 4-methylcatechol and acetylsalicylic acid on arachidonic acid- and collagen-triggered platelet aggregation. (**A**,**B**) Aggregation was induced by 200 µM of arachidonic acid. (**C**,**D**) Aggregation was triggered by 1 µg/mL of collagen. (**A**,**C**) Relative aggregation (each value represents the percent inhibition of platelet aggregation vs. the control sample with dimethyl sulfoxide (DMSO) and the corresponding inducer). (**B**,**D**) Absolute values measured by the area under the curve (AUC). * *p* < 0.05, ** *p* < 0.01, *** *p* < 0.001. The data are shown as the median ± 95% confidence intervals. ASA—acetylsalicylic acid 4-MC—4-methylcatechol.

The strong antiplatelet activity of 4-MC was confirmed in this group. The effect of 10 µM of 4-MC lay between the effect of 30 µM and 70 µM of ASA on AA-triggered platelet aggregation. This was seen both when relative effects (the percent inhibition of platelet aggregation in each donor vs. the solvent) and absolute effects (the area under the curve) were compared (Figure 1A,B). ASA at the lower concentration decreased platelet aggregation to 75.0 ± 19.6% of the control values, while ASA at the higher concentrations decreased it to 40.0 ± 18.1%, and 4-MC decreased it to 55.2 ± 22.2%. When the areas under the curves were compared, 4-MC was by ~26% more active than 30 µM of ASA, while 70 µM of ASA was ~30% more potent than 10 µM of 4-MC.

In the case of collagen, higher concentrations were selected based on the lower effect of both ASA and 4-MC on this cascade. The effect of 70 µM ASA lay between the effect of 20 and 70 µM of 4-MC. The differences were more apparent in the case of relative effects (Figure 1C). In terms of AUC, there was only a numerical, but not a significant, difference between the effects of ASA and 4-MC (Figure 1D). In relative terms, the lower concentration of 4-MC decreased platelet aggregation to 90.0 ± 11.7%, while the higher concentration decreased it to 65.0 ± 15.6%. The effect of ASA was 74.0 ± 15.6% of the control values. The effect of 70 µM of 4-MC was ~27% more expressed than that of 20 µM, while ASA 70 µM was ~17% more potent than 20 µM of 4-MC but ~12% less potent than the equimolar concentration of the same compound.

**Figure 2:**
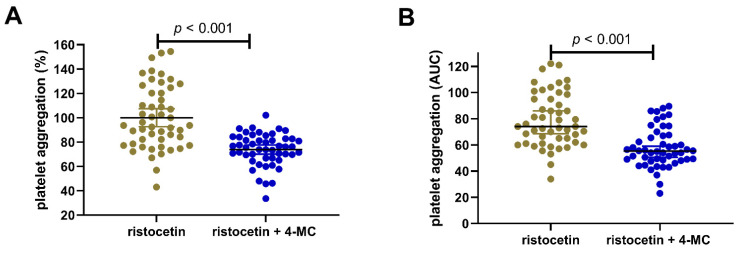
Ristocetin-induced platelet aggregation and the effect of 4-methylcatechol. (**A**) Relative changes vs. the corresponding blank (DMSO + ristocetin), (**B**) Absolute values are expressed as the area under the curve (AUC). The data are shown as the mean ± 95% confidence intervals in the case of relative values and the median ± 95% confidence intervals in the case of AUC. The concentration of 4-MC was 240 μM. 4-MC—4-methylcatechol.

As the last test to confirm the efficacy of 4-MC, the effect on the von Willebrand factor cascade was investigated. This pathway is stimulated by ristocetin, and our results showed that 4-MC was able to block it partially. In this group, 4-MC at a high concentration of 240 µM blocked this aggregation to 73.9 ± 13.3% of the control values (Figure 2). 

There were no significant relationships between the response to 4-MC and different anthropometric (height, weight, BMI) and biochemical–metabolic values (cholesterol, glucose, triglycerides) (Supplementary Materials Table S3). There was also no correlation between age and the response to 4-MC. Absolute platelet aggregation responses to 4-MC were numerically higher in women than in men, but the data did not reach statistical significance (Supplementary Materials Figure S1).

**Figure 3:**
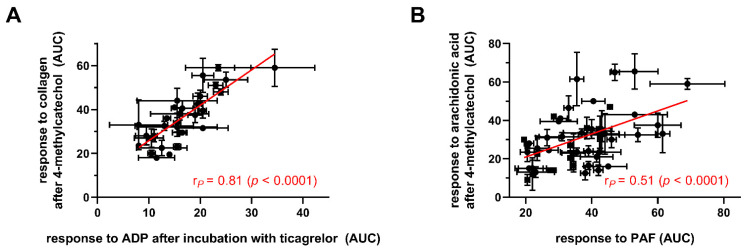
Examples of strong associations between responses to 4-methylcatechol and other inducers/antagonists. (**A**): ADP after ticagrelor incubation with collagen (1 μg/mL) after 4-methylcatechol (70 μM) treatment, (**B**): Platelet-activating factor-16 (PAF) with arachidonic acid (200 μM) after 4-methylcatechol (10 μM) incubation. ADP—adenosine-5’-diphosphate.

The response to 4-MC could be predicted in many cases from other aggregatory responses (Table 2, Figure 3).

The strongest correlations were found for relationships between the response to 4-MC (20 µM) in collagen-induced platelet aggregation and responses to solely collagen and AA or the response to collagen with ASA as the antiplatelet agent. The responses to 4-MC were apparently linearly related to the magnitude of responses to AA, collagen or ristocetin or to the effect of ASA. While these relationships were expected, the responses to 4-MC also correlated with other unrelated pathways (in particular to ticagrelor + ADP or PAF, Figure 3).

In the second part, the mechanism of action was investigated. In our previous paper [7], we showed that 4-MC did not significantly affect cyclooxygenase 1, and even though it had an effect on thromboxane synthase, this inhibition was not considered clinically important since it was achieved at a relatively high concentration of 100 μM. Subsequently, 4-MC did not inhibit thromboxane binding to its receptors or its intracellular trafficking, and its antiplatelet effects were not abolished by addition of extracellular calcium or other metal ions but were apparently dependent on intracellular calcium signaling. 

**Figure 4:**
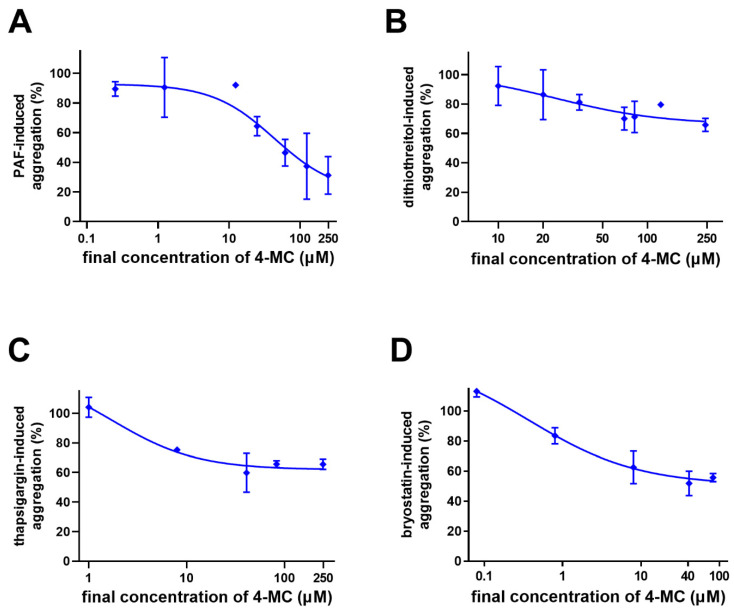
Investigation of the mechanism of the action of 4-methylcatechol (4-MC). In each of the experiments, 4-MC was incubated for 3 min with either blood or platelet-rich plasma (PRP), and aggregation was triggered afterward by the addition of an inducer. (**A**) The effect of 4-MC on platelet-activating factor 16 (PAF)—induced aggregation; aggregation was initiated by adding PAF (the final concentration in PRP was 40 nM). (**B**) The effect of 4-MC on dithiothreitol-induced aggregation in whole human blood; aggregation was initiated by adding dithiothreitol at a final concentration of 410 µM. (**C**) The effect of 4-MC on thapsigargin-induced aggregation in whole human blood; aggregation was initiated by adding thapsigargin (the final concentration varied from 83 to 165 nM according to calibration in each individual donor). (**D**) The effect of 4-MC on bryostatin (8 nM)—induced aggregation in human whole blood.

As ADP and thrombin (TRAP) pathways are not influenced by 4-MC, we turned our focus to other pathways. The effect of 4-MC was studied on PAF-, dithiothreitol-, thapsigargin- and bryostatin 1-induced aggregation (Figure 4). As in the population group study, an effect on ristocetin-induced aggregation was also found. 

**Figure 5:**
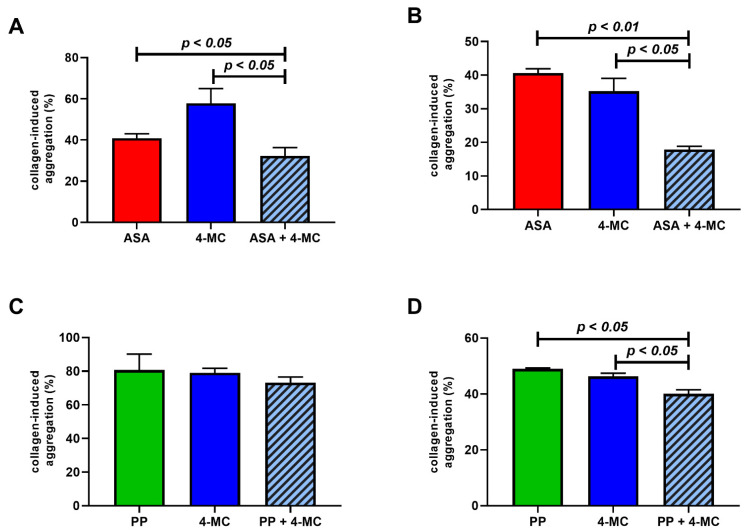
Investigation of the coadministration of 4-methylcatechol (4-MC) with known inhibitors on arachidonic acid-related aggregation pathways. (**A**) The effect of 4-MC, ASA and their combination on collagen-induced aggregation; blood was incubated with 5 μM of 4-MC, 53 μM of ASA and their combination, and aggregation was triggered by adding of collagen (final concentration of 0.5 μg/mL). (**B**) The effect of 4-MC, ASA and their combination on collagen-induced aggregation; blood was incubated with 80 μM of 4-MC, 53 μM of ASA and their combination, and aggregation was triggered by adding collagen (0.4 µg/mL). (**C**) The effect of 4-MC, pyrrophenone (PP) and their combination on collagen-induced aggregation; blood was incubated with 5 μM of 4-MC, 200 nM of PP and their combination, and aggregation was triggered by collagen (0.16 µg/mL). (**D**) The effect of 4-MC, PP and their combination on collagen-induced aggregation; blood was incubated with 123 μM of 4-MC, 2 μM of PP and their combination, and aggregation was triggered by adding collagen (0.16 µg/mL).

As ristocetin is known to mimic the proaggregatory effect of the von Willebrand factor, which initiates the aggregation cascade in cooperation with collagen, we returned back to the AA-related cascade since these pathways are closely related. In the first step, the possible potentiation of the antiplatelet effect of ASA by 4-MC was tested in two settings: (a) with a low but active concentration of 4-MC (5 μM) to see if the effect of a relatively high concentration of ASA could be enhanced; (b) with a high concentration of 4-MC (80 μM) added to a high concentration of ASA (80 μM, a further increase in this concentration does not increase the antiplatelet effect [7]) to see if the maximal effect of ASA could be improved. In both settings (Figure 5A,B), the addition of 4-MC improved the effect of ASA, emphasizing that their mechanisms of action are not identical. 

In order to study whether the release of AA, which is triggered by collagen, could be involved, pyrrophenone, an inhibitor of phospholipase A_2_, was tested. Our results show that pyrrophenone was more active at the molecular level, and significant effects were already observed at hundreds of nM. At such a low concentration of pyrrophenone, 4-MC at a low concentration of 5 μM did not improve the effect of pyrrophenone. However, when high concentrations were used, the maximal effect of pyrrophenone was slightly but significantly improved by 4-MC (Figure 5C,D). These results again suggested a different mechanism of action. 

**Figure 6:**
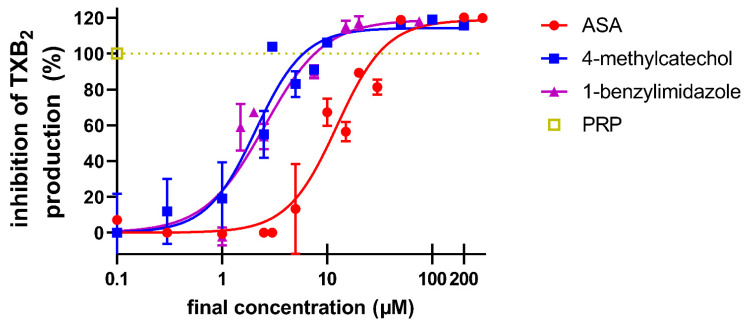
The effect of the tested compounds on the synthesis of thromboxane A_2_ from arachidonic acid. Platelet-rich plasma (PRP) treated with thromboxane receptor antagonist terutroban at a final concentration of 2 µM in order to hinder the activation of platelet aggregation was incubated with the tested compounds for 3 min. The reaction was initiated by adding arachidonic acid (AA) at a final concentration of 100 µM and terminated after 7 min by adding a cold ethylenediaminetetraacetic acid disodium salt solution at a final concentration of 1 mM. PRP—a blank sample treated under the same conditions without the addition of AA. TXB_2_—thromboxane B_2_.

Since there are no other known participating factors in the AA-based cascade, the last pathway we tested was the synthesis of thromboxane A_2_ from AA. As mentioned above, 4-MC was not able to significantly block either cyclooxygenase-1 or thromboxane synthase. Therefore, we directly added the substrate AA into platelets and measured the production of the final product of thromboxane A_2_. Both 4-MC and a known inhibitor of thromboxane synthase, 1-benzylimidazole, blocked the formation of thromboxane A_2_. ASA, as the cyclooxygenase-1 inhibitor, was logically also active, but to a lesser extent (Figure 6). Taking into account these and our previous results, we suppose that 4-MC is able to uncouple the cyclooxygenase-1 and thromboxane synthase collaboration in the synthesis of thromboxane A_2_, and this could explain the observed effects of 4-MC on several, but not all aggregatory pathways.

## 4. Discussion

Flavonoid and other polyphenol-rich diets have been attracting significant interest due to their claimed positive cardiovascular effects in humans. These effects could be based on reported antiplatelet, vasodilatory and potentially also other effects (e.g., the inhibition of reactive oxygen species forming enzymes, interaction with trace metals and direct radical scavenging effects) [20]. Understanding the relationship between flavonoids and their positive effects on humans is, however, burdened by the fact that the oral absorption of flavonoids is low. The current research seems to offer an explanation that lies in the metabolism of flavonoids and other phenolic compounds with low bioavailability by human intestinal microbiota into small phenolic compounds. These small phenolic compounds are then absorbed, and some of them have been shown to possess significant biological effects. One of these metabolites that has been largely investigated is 4-MC. Indeed, plasma levels of its sulfate conjugate reached a concentration of 3.5 μM in plasma after the administration of cranberry juice, which is clearly more than the vast majority of flavonoids and other parent phenolic substances [14,21].

For this reason, we focused on 4-MC and found that this compound is a fairly potent vasodilator and also an antiplatelet drug with an IC_50_ of about 3 μM in AA-based aggregation [7,22]. This result was, however, based on a limited number of very young adults. Regardless, the results from the present study apparently confirm the previously observed outcome, and on a molecular basis, 4-MC was shown to be more potent than ASA both in AA- and collagen-triggered platelet aggregation. This result is encouraging for future studies with patients with cardiovascular risk factors.

In the second part of the study, future insight into the mechanism of action was obtained. In addition to previously tested inducers, other compounds stimulating specific pathways were selected. PAF is an important messenger for many different cell populations. It acts on platelets through its PAF-1 receptor, which is G_q_ coupled. G_q_ receptors cause both an increase in Ca^2+^ and the activation of protein kinase C (PKC) through inositol triphosphate (IP_3_) and diacylglycerol release, respectively [23,24]. As 4-MC previously blocked Ca^2+^-triggered platelet aggregation by calcium ionophore [7], it was of interest to test whether it could also block this pathway. Indeed, 4-MC also inhibited PAF-induced aggregation. Since the extent of this inhibition was not very pronounced, interference at the receptor level can be excluded. In addition to the mentioned Ca^2+^ signaling, an effect on PKC activation may also be involved. In order to confirm or exclude the involvement of PKC, bryostatin, an extremely potent modulator of PKC, was included in our testing. Bryostatin activates this enzyme directly in platelets and causes Ca^2+^ influx and platelet aggregation [25,26]. Seeing that 4-MC was active also in this case, we supposed it might affect PKC directly, as bryostatin does not require diacylglycerol for its effect. In order to observe whether platelet aggregation mediated by an increase in calcium at intracellular levels from other pathways could also be blocked, thapsigargin, an inhibitor of sarco/endoplasmic reticulum Ca^2+^-ATPase (SERCA), was employed. Thapsigargin increases intracellular calcium levels by blocking this ATPase, which leads to subsequent platelet activation and aggregation [27,28,29]. Our data clearly showed a decrease in thapsigargin-induced aggregation, again emphasizing that the effect of 4-MC is linked with the interference of Ca^2+^ signaling. 

The last step of platelet aggregation is associated with the activation of the integrin IIb/IIIa, and in this case, both PKC and Ca^2+^ participate in this pathway. Glycoprotein IIb/IIIa can be activated by dithiothreitol, which modifies the protein thiol and sulfide groups required for its function [30,31]. Dithiothreitol-induced aggregation is not associated with any platelet shape change and granule secretion and is not inhibited by prostaglandin E_1_ [30,31]. 4-MC inhibited dithiothreitol-induced aggregation only partially; therefore, interference directly at this receptor is again unlikely. Considering that dithiothreitol is not a “full” inducer, i.e., it does not evoke a response of such a magnitude as either ADP or collagen, it is most probable that 4-MC interferes with intracellular downstream signaling from these receptors. The suggested interaction between Ca^2+^ levels and PKC would provide an explanation for this phenomenon.

However, as the inhibitory effect of 4-MC on these cascades was not complete or only nearly complete, the major mechanism of action is apparently based on a different mechanism. Because the most potent effect of 4-MC was observed on AA-based platelet aggregation, subsequent research was targeted toward this pathway. 

Pyrrophenone, a specific cytosolic phospholipase-2 inhibitor, blocks the release of AA from membranes and hence inhibits the corresponding AA-based cascade at its beginning [32,33]. We did not observe a large improvement when it was combined with 4-MC. This further supported the hypothesis that the mechanism of 4-MC is based on a downstream target.

When 4-MC was combined with ASA, there was a significant decrease in platelet aggregation, even at the lower concentrations of these compounds. ASA inhibits cyclooxygenase 1, which is the only form of cyclooxygenase present in platelets. This result could offer several possible explanations; 4-MC either acts completely independently on the AA-based cascade, or it interferes with the next step, the transformation of prostaglandin H_2_ (PGH_2_). As we did not observe a significant inhibition of thromboxane synthase or cyclooxygenase-1 with 4-MC, nor did 4-MC affect thromboxane (U-46619)-induced platelet aggregation, the answer was not clear [7]. In order to check whether 4-MC could interfere with the whole cyclooxygenase-thromboxane synthase mediated formation of thromboxane A_2_, the levels of this product were measured after the incubation of platelets with AA. Interestingly, the effect of 4-MC was similar to that of a known thromboxane synthase inhibitor, 1-benzylimidazole. More importantly, the effect was about 1 order of magnitude more potent than that of ASA, which apparently reflects the higher potency of 4-MC observed previously with AA [7]. Both cyclooxygenase 1 and thromboxane synthase are localized in the endoplasmic reticulum (in platelets known as dense tubular system). The same organelle is also important storage of calcium, so the effect of 4-MC, which in some way interrupts the coupling of these two enzymes, might also be related to the observed interaction with calcium signaling [34]. We cannot currently fully confirm this hypothesis as there are no data reporting in detail how cyclooxygenase-1 collaborates with thromboxane synthase. 

Our recent studies confirmed the lack of toxicity of 4-MC towards platelets and red blood cells [7,35]. However, it should also be mentioned that previous studies have shown that 4-MC can have both protective and cytotoxic properties, depending on the cell type, dose and level of oxygen in the culture. Contrarily, in neural cell cultures, 4-MC improved their survival and promoted their growth and the expression of neural growth factors. Interestingly, it seemed to promote the apoptosis of cancer cell lines while insignificantly affecting normal cell types, as seen with melanoma or liver cells [36,37,38,39,40,41,42,43].

An interesting finding of this study is the correlations between the degree of the 4-MC antiplatelet effect and the magnitude of the response to other aggregation inducers with or without standard antiplatelet drugs (Table 2, Figure 3). Some of them were really unexpected as they are unrelated pathways, e.g., 4-MC has no effect on ADP-platelet aggregation [7], but there was a strong relationship to inhibition of ADP-triggered pathway by ticagrelor. The interpretation of these data is hence not easy, but it seems that platelets from generally healthy donors are reacting somehow predictably, so if the platelet response to one agonist is strong/weak, the response to another trigger is also similarly high/low, and the degree of inhibition by 4-MC and standard drugs is corresponding. It would be of real clinical interest to see if this relationship will also be kept in blood from patients suffering from different metabolic or cardiovascular diseases, as at least in diabetic persons, a substantial portion of the patients is resistant or less responding to ASA [44,45], but they might continue to respond to 4-MC. A study testing this is currently ongoing in our laboratory.

## 5. Conclusions

**Figure 7:**
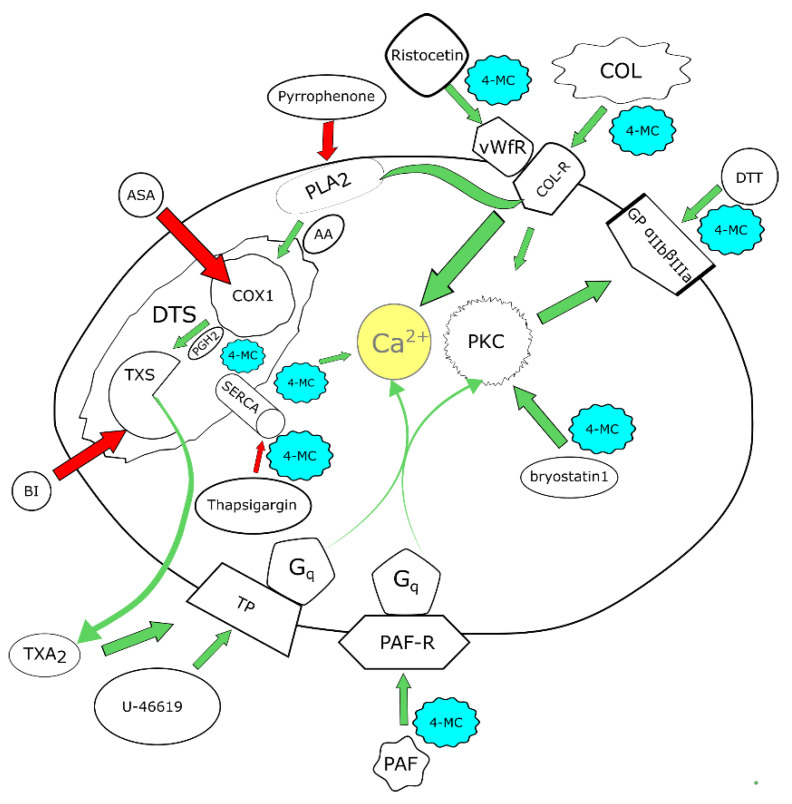
Summary of the tested effects of 4-methylcatechol (4-MC) in the platelet. The red arrows show inhibition, while the green arrows show activation. Blue cloudlets with 4-MC show the places where 4-MC was active, e.g., where platelet aggregation triggered by the used inducer was affected by 4-MC. ASA—acetylsalicylic acid, COX1—cyclooxygenase 1, AA—arachidonic acid, PLA_2_—phospolipase A_2_, vWfR—von Willebrand factor receptor, COL—collagen, COL-R—collagen receptors, DTT—dithiothreitol, PKC—protein kinase C, PAF—platelet-activating factor, PAF-R—platelet activating factor receptor, SERCA—sarco/endoplasmic reticulum Ca^2+^-ATPase, TP—thromboxane receptor, TXA_2_—thromboxane A_2_, BI—1-benzylimidazole, PGH_2_—prostaglandin H2, DTS—dense tubular system, TXS—thromboxane synthase, GP αIIbβIIIa— glycoprotein α_IIb_β_IIIa_, G_q_— Gq protein, U-46619—9,11-dideoxy-9α,11α-methanoepoxy prostaglandin F2α.

In summary, this study confirmed the antiplatelet effects of 4-MC in a relatively large group of healthy blood donors. Furthermore, the results obtained support the hypothesis that 4-MC not only acts on the AA-based cascade but interferes with several aggregation pathways (Figure 7). The major mechanism relies likely on the disruption of cyclooxygenase-1-thromboxane synthase coupling.

## Figures and Tables

**Table 1:** Aggregation inducers and inhibitors in cross-sectional part of this study.

Tested Compound	Final Concentration	Inducer	Final Concentration ^1^
4-MC	240 µM	ristocetin	400 µM
	70 µM	collagen	1 µg/mL
	20 µM		
	10 µM	AA	200 µM
ASA	70 µM	collagen	1 µg/mL
		AA	200 µM
	30 µM	AA	200 µM
ticagrelor	0.5 µM	ADP	5 µM
vorapaxar	1 and 5 µM	TRAP	10 µM
		U-46619	80 nM
		PAF	20 nM

^1^ Final concentrations in the aggregation cuvettes. 4-MC—4-methylcatechol, ASA—acetylsalicylic acid, AA—arachidonic acid, ADP—adenosine-5’-diphosphate, TRAP—thrombin receptor agonist peptide-6, U-46619—9,11-dideoxy-9α,11α-methanoepoxy prostaglandin F2α, PAF—platelet-activating factor-16.

**Table 2:** Relationships between the responses to 4-methylcatechol and other aggregation responses (expressed as AUC).

	4-MC 10 μM + AA 200 μM	4-MC 20 μM + Collagen 1 μg/mL	4-MC 70 µM + Collagen 1 µg/mL	4-MC 240 µM + Ristocetin
AA 60 µM	0.67 ***	0.39 **	n.s.	0.26 (*p* = 0.07)
AA 200 µM	0.36 **	0.78 ***	0.47 **	0.60 ***
collagen 0.16 µg/mL	0.55 ***	0.61 ***	0.63 ***	0.29 *
collagen 1 µg/mL	0.29 *	0.87 ***	0.60 ***	0.55 ***
ADP	0.54 ***	0.63 ***	0.55 **	0.58 ***
ristocetin	0.57 ***	0.65 ***	0.34 (*p* = 0.06)	0.71 ***
TRAP	0.30 *	0.65 ***	n.s.	0.49 ***
U-44619	0.43 **	0.60 ***	n.s.	0.45 **
PAF	0.51 ***	0.51 ***	n.s.	0.52 ***
ASA 30 μM + AA 200 μM	0.69 ***	0.50 ***	0.35 *	0.29 *
ASA 70 μM + AA 200 μM	0.60 ***	0.28 *	n.s.	n.s.
ASA 70 μM + collagen 1 μg/mL	0.32 *	0.81 ***	0.69 ***	0.47 ***
ticagrelor + ADP	0.36 *	0.59 ***	0.81 ***	0.36 *

The data show the Pearson correlation coefficients. n.s.—nonsignificant, * *p* < 0.05, ** *p* < 0.01, *** *p* < 0.001. The highest coefficients are highlighted in red. Nearly significant associations are in grey. AUC—area under the curve.

## Supplementary Materials

The following supporting information can be downloaded at: https://www.mdpi.com/article/10.3390/nu14224798/s1, Figure S1: Gender differences in responses to 4-methylcatechol. Table S1: Summary of diagnoses of the blood donors. Table S2: Summary of medications taken by the blood donors at the time of blood draw. Table S3: Level of statistical significance of relationships between biochemical and anthropometric parameters with aggregatory responses to 4-methylcatechol.
