# Supplementary material for: The Antiplatelet Effect of 4-Methylcatechol in a Real Population Sample and Determination of the Mechanism of Action
Source: Nutrients. 2022 Nov 13;14(22):4798. doi: 10.3390/nu14224798 (PMC9694226; doi:10.3390/nu14224798)
Supplement: Supplementary file 1 [file nutrients-14-04798-s001.zip › nutrients-1981216-supplementary.pdf]

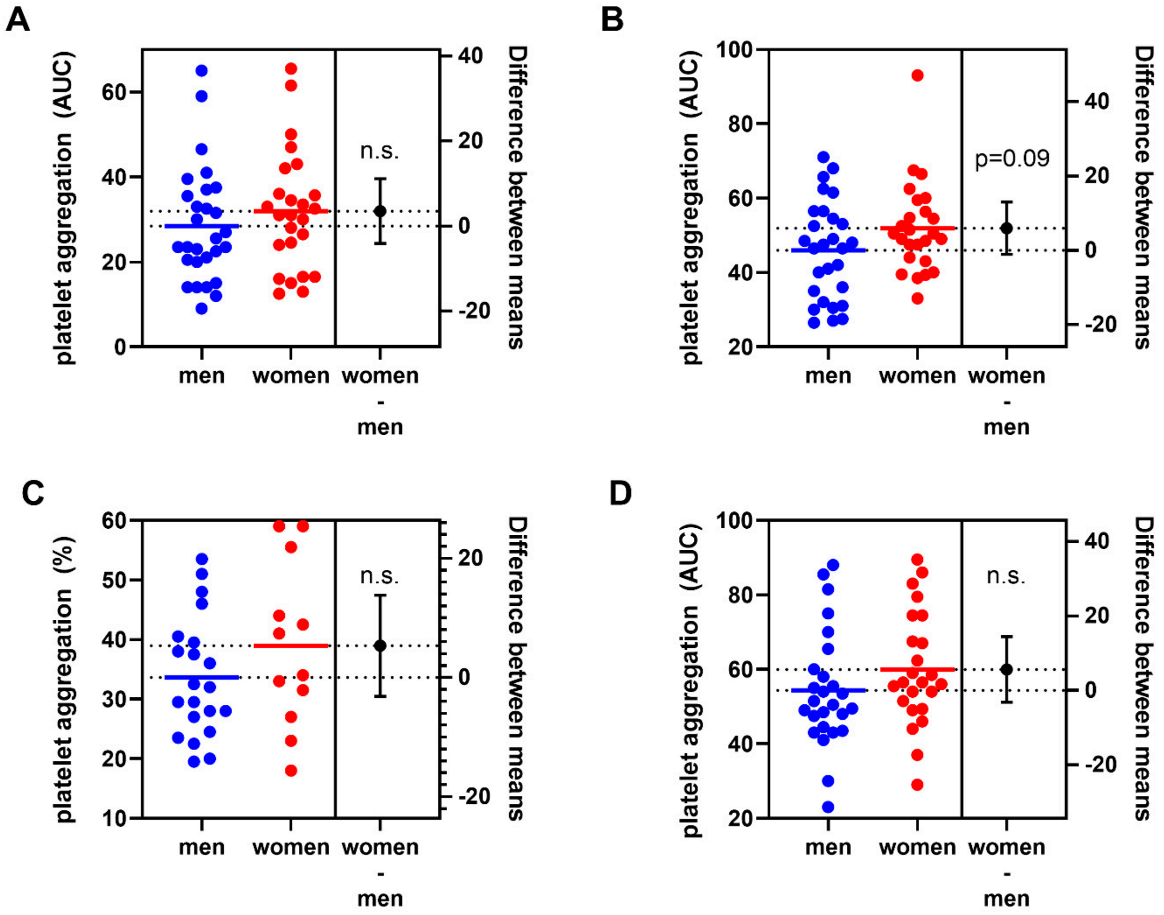

Figure S1. Gender differences in responses to 4-methylcatechol. **A:** 4-methylcatechol (10 μM) + arachidonic acid (200 μM), **B**: 4-methylcatechol (20 μM) + collagen (1 μg/mL), **C**: 4-methylcatechol (70 μM) + collagen (1 μg/mL), **D**: 4-methylcatechol (240 μM) + ristocetin (400 μM). n.s., nonsignificant.

Table S1. Summary of diagnoses of the blood donors.

Condition	Donors–number (%)
hypertension	10 (19%)
allergy	7 (13%)
hypothyreosis	6 (11%)
asthma	2 (4%)
gastroesophageal reflux disease	2 (4%)
chronic glomerulonephritis	1 (2%)
gout	1 (2%)
hepatopathy	1 (2%)
nephrolithiasis	1 (2%)
obesity	1 (2%)
prediabetes	1 (2%)
glaucoma	1 (2%)

Table S2. Summary of medications taken by the blood donors at the time of blood draw.

Medication	Donors–number (%)
antihistamine	8 (15%)
thyroid hormone	6 (11%)
angiotensin receptor II antagonist	4 (8%)
calcium channel blocker	4 (8%)
proton pump inhibitor	3 (6%)
inhalatory corticoid	2 (4%)
inhalatory β_2-agonist	2 (4%)
allopurinol	1 (2%)
intestinal antiflogistic	1 (2%)
bile acid	1 (2%)
prostaglandin analogue (topical)	1 (2%)

Table S3. Level of statistical significance of relationships between biochemical and anthropometric parameters with aggregatory responses to 4-methylcatechol.

	4-MC 10 µM + AA 200 µM	4-MC 20 µM + collagen 1 µg/mL	4-MC 70 µM + collagen 1 µg/mL	4-MC 240 µM + ristocetin
height	0.65	0.18	0.09	0.19
weight	0.53	0.45	0.56	0.51
BMI	0.59	0.92	0.75	0.11
glucose	0.14	0.79	0.35	0.92
HDL-cholesterol	0.98	0.14	0.72	0.39
LDL-cholesterol	0.57	0.68	0.79	0.56
non HDL cholesterol	0.78	0.58	0.67	0.59
total cholesterol	0.77	0.99	0.76	0.36
triglycerides	0.92	0.89	0.67	0.92

Data in the table shows level of the statistical significance (p). There were no significant relationships; the p values are relatively high suggesting no associations with exception of the relationship between the height and the response to collagen after 4-methylcatechol (4-MC) treatment ($p = 0.09$). ASA 30 + AA 200—acetylsalicylic acid 30 µM + arachidonic acid 200 µM, ASA 70 + AA 200—acetylsalicylic acid 70 µM + arachidonic acid 200 µM, ASA 70 + collagen 1—acetylsalicylic acid 70 µM + collagen 1 µg/mL. BMI, body mass index; HDL, high-density lipoprotein; LDL, low-density lipoprotein.
